# Trends in Canadian prescription drug purchasing: 2001–2020

**DOI:** 10.1186/s40545-022-00420-4

**Published:** 2022-03-17

**Authors:** Mark Hofmeister, Ashwinie Sivakumar, Fiona Clement, Kaleen N. Hayes, Michael Law, Jason R. Guertin, Heather L. Neville, Mina Tadrous

**Affiliations:** 1 Department of Community Health Sciences, University of Calgary, Calgary, AB Canada; 2 O’Brien Institute for Public Health, University of Calgary, Calgary, AB Canada; 3 Leslie Dan Faculty of Pharmacy, University of Toronto, Toronto, ON Canada; 4 Dalla Lana School of Public Health, University of Toronto, Toronto, ON Canada; 5 Centre for Health Services and Policy Research, School of Population and Public Health, University of British Columbia, Vancouver, BC Canada; 6 Axe Santé Des Populations et Pratiques Optimales en Santé, Centre de Recherche du CHU de Québec-Université Laval, Québec, Canada; 7 Department of Social and Preventive Medicine, Université Laval, Québec, Canada; 8 Queen Elizabeth II Health Sciences Centre, Nova Scotia Health Authority, Halifax, NS Canada; 9 Women’s College Research Institute, Women’s College Hospital, 76 Grenville St., Toronto, ON Canada; 10 ICES, Toronto, ON Canada

## Abstract

**Background:**

In 2019, more than $34.5 billion was spent on prescription drugs in Canada. However, little is known about the distribution of this spending across medications and settings (outpatient and inpatient) over time. The objective of this paper is to describe the largest expenditures by medication class over time in inpatient and outpatient settings. This information can help to guide policies to control prescription medication expenditures.

**Methods:**

IQVIA’s Canadian Drugstore and Hospital Purchases Audit data from January 1, 2001, to December 31, 2020, were used. In this dataset, purchasing was stratified by outpatient drugstore and inpatient hospital. Spending trajectories in both settings were compared to total expenditure over time. Total expenditure of the 25 medications with the largest expenditure were compared over time, stratified by setting. Nominal costs were used for all analysis.

**Results:**

In 2001, spending in the outpatient and inpatient settings was greatest on atorvastatin ($467.0 million) and erythropoietin alpha ($91.2 million), respectively. In 2020, spending was greatest on infliximab at $1.2 billion (outpatient) and pembrolizumab at $361.6 million (inpatient). Annual outpatient spending, although increasing, has been growing at a slower rate (5.3%) than inpatient spending (7.0%). In both settings, spending for the top 25 medications has become increasingly concentrated on biologic agents, with a reduction in the diversity of therapeutic classes of agents over time.

**Discussion:**

Identification of the concentration on spending on biologic agents is a key step in managing costs of prescription medications in Canada. Given the increases in spending on biologic agents over the last 20 years, current cost-control mechanisms may be insufficient. Future research efforts should focus on examining the effectiveness of current cost-control mechanisms and identifying new approaches to cost control for biologic agents.

**Supplementary Information:**

The online version contains supplementary material available at 10.1186/s40545-022-00420-4.

## Background

In 2019, total prescribed drug spending in Canada was 34.3 billion, of which public drug program spending accounted for 43.6% of spending at $15.0 billion [1]. Outside of the public sector, spending by private insurers was $12.7 billion, with the remaining $6.8 billion paid for by Canadian households [2 as cited in 1]. Prescription drug spending is estimated to increase from 4.2% to 4.6% per year from 2021 to 2023, for total spending of approximately $37.2 billion in 2023 [3]. Often, estimates of public spending on prescription drugs do not include drugs dispensed in hospitals or those funded outside of public drug plans such as provincial programs [1]. Available estimates of drug spending in Canada often focus on payer-specific outpatient expenditures, neglecting inpatient drug spending [3]. Tadrous et al. [3] demonstrated that not only are the trajectories of spending different in the outpatient and inpatient setting but that the lack of attentiveness to the inpatient setting has resulted in larger relative increases in the inpatient expenditure over time compared to the growth in outpatient expenditure.

One approach to effective cost-control strategies for the outpatient and inpatient sectors could be to implement specific cost-control mechanisms such as generic substitution, mandatory biosimilar substitution, or lowest cost alternative pricing. However, data are needed to identify patterns of expenditure over time to identify areas where cost control may be needed. The American Society of Health System Pharmacists (ASHP) guidelines suggest that a first step in managing costs of medications should be to collect and review drug purchase data, focusing on high-priority agents [4]. Recently, Tadrous et al. [3] described how spending on the top 25 drugs accounted for 52.9% and 26.0% of total spending in outpatient and inpatient sectors, respectively. In order to begin to manage the costs of medications, high-priority agents must be identified.

Therefore, the objective of this paper is to identify medications with the highest expenditure and describe changes in their spending trends over time, in both outpatient and inpatient sectors. This information could then be used to develop targeted cost-control mechanisms for individual therapeutic classes relevant to the outpatient and inpatient sector.

## Methods

Data from IQVIA’s Canadian Drugstore and Hospital Purchases Audit from January 1, 2001 to December 31, 2020 were used, which was the most recent complete calendar year at the time of writing. These data include total expenditure of each pharmaceutical product purchased by drugstores (outpatient) and hospitals (inpatient), from a sample of individual outlets within outpatient and inpatient sectors in each province and territory [5]. Sample data are projected to both inpatient and outpatient settings to estimate total spending in Canada [5]. These data are unique for their stratification into outpatient and inpatient sectors. Regardless of the source of purchase, either directly from manufacturers or through wholesalers, markups; discounts, rebates, and dispensing fees that would be paid by patients are excluded [5]. Aggregate data were obtained outlining total annual spending, stratified by inpatient and outpatient setting. Specific data were obtained for 25 highest expenditure drugs, ranked in descending order by total expenditures.

Total drug spending and spending stratified by setting were analyzed descriptively. Changes in annual growth rates were compared for total drug spending and by setting. Annual growth rates were also estimated for the top 25 drugs in each sector. The proportion of total spending by sector for the top 25 drugs was calculated. This analysis has selected the top 25 agents within each sector to focus on, to describe further concentration/diffusion patters within those agents. Within sectors, spending on the top 25 agents was compared to total drug spending, as the proportion of spending within each sector. For 2001 and 2020, spending on specific agents was explored.

The top 25 drugs present in inpatient and outpatient sectors were categorized into single therapeutic classes by an expert pharmacist. Therapeutic classifications were aligned with World Health Organization Anatomical Therapeutic Chemical classifications, with further delineation added as required. Spending across therapeutic groups for top 25 drugs is compared for selected years. Consistent with reports generated by the Canadian institute for Health Information [6], all costs are presented in nominal Canadian dollars.

## Results

### Growth in spending by sector

**Fig. 1:**
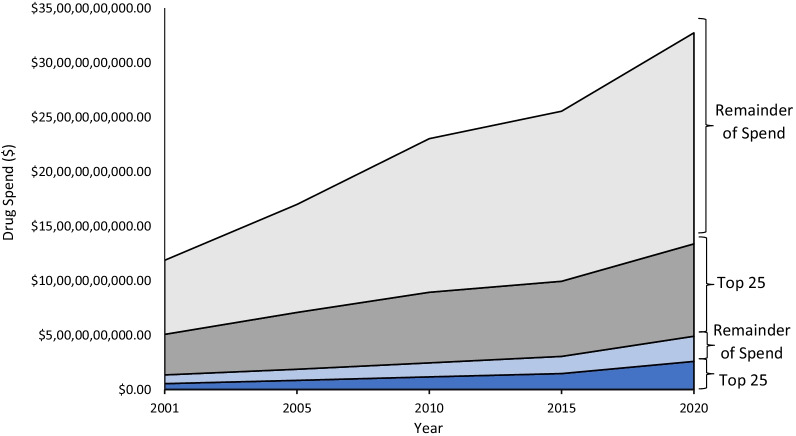
Outpatient and inpatient drug spending in selected years. Grey represents outpatient drug spending, and blue represents inpatient drug spending

From 2001 to 2020, total drug spending in Canada nearly tripled, increasing from $11.9 billion to $32.7 billion (average annual growth of 5.5% per year). Growth in outpatient drug spending was lower, with an average annual increase of 5.3% from $10.5 billion in 2001 to $27.8 billion in 2020. Over the same period, the average annual increase in inpatient drug spending was 7.0%, from $1.3 billion in 2001 to $4.9 billion in 2020 (Fig. 1).

In 2001, spending for the top 25 drugs purchased for use in outpatient settings in Canada was 31.4% of total drug spending. The proportion spent on the top 25 drugs purchased in the outpatient sector decreased steadily to 25.9% in 2020. Within the outpatient setting, spending on the top 25 medications increased at an average rate of 4.4% per year. In 2001 outpatient spending for the top 25 medications by expenditure was $3.7 billion, and in 2020, $8.5 billion. In 2001, the top 25 drugs by spending made up 35.4% of total outpatient purchases by drugstores. In 2020, the top 25 drugs by spending had decreased to make up 30.5% of total outpatient purchases.

Similarly, spending for the top 25 drugs purchased by inpatient settings in Canada in 2001 was 4.59% of total drug spending. The proportion spent on the top 25 drugs purchased in inpatient settings increased steadily to 7.9% in 2020. Within inpatient spending, growth was concentrated in the top 25 medications, with an average annual growth rate of 8.5%: from $545 million in 2001 to $2.6 billion in 2020. In 2001, the top 25 drugs made up 40.5% of total inpatient drug purchases. In 2020, the top 25 drugs had increased to make up 52.9% total inpatient drug purchases.

### Top 25 drugs ranked by expenditure in outpatient and inpatient sectors

Within the top 25 drugs purchased in the outpatient sector in 2001, spending was greatest on atorvastatin at $467.0 million, followed by omeprazole at $408 million. In third place was amlodipine, with total spend of $207.9 million in 2001. In 2020, the drug with the greatest spend in the outpatient sector was infliximab, at $1.2 billion, followed by adalimumab at $970.1 million, and ustekinumab at $527 million (Table 1).

**Table 1:** Top 25 drugs ranked by expenditure, highest to lowest, in 2001 and 2020

Outpatient sector		Inpatient sector	
2001	2020	2001	2020
1. Atorvastatin	1. Infliximab	1. Erythropoietin alpha	1. Pembrolizumab
2. Omeprazole	2.Adalimumab	2. Alteplase	2. Nivolumab
3. Amlodipine	3. Ustekinumab	3. Cyclosporine	3. Daratumumab
4. Paroxetine	4. Aflibercept	4.Irinotecan	4.Rituximab
5. Simvastatin	5. Apixaban	5. Clozapine	5. Trastuzumab
6. Fluticasone	6. Metformin–sitagliptin	6. Pamidronic acid	6. Pertuzumab–Trastuzumab
7. Celecoxib	7. Semaglutide	7. Paclitaxel	7. Ibrutinib
8. Ramipril	8. Ranibizumab	8. Olanzapine	8. Durvalumab
9. Rofecoxib	9. Methylphenidate	9. Ciprofloxacin	9. Bevacizumab
10. Enalapril	10. Etanercept	10. Docetaxel	10. Vaccine, Pneumococcal conjigate
11. Olanzapine	11. Rivaroxaban	11. Filgrastim	11. Palbociclib
12. Venlafaxine	12. Sofosbuvir–velpatasvir	12. Ceftriaxone	12. Haemagglutinin (non-specific)
13. Pantoprazole	13. Budesonide–formoterol	13. Enoxparin	13. Darbepoetin alfa
14. Diltiazem	14. Empaglifozin	14. Rituximab	14. Ipilimumab
15. Pravastatin	15. Lisdexamfetamine	15. Trastuzumab	15. Osimertinib
16. Nifedipine	16. Insulin glargine	16. Abciximab	16. Nusinersen
17. Lansoprazole	17. Ibrutinib	17. Goserelin	17. Vaccine: HPV Type-6,11,16,18,3
18. Lisinopril	18. Rosuvastatin	18. Sevoflurane	18. Erythropoietin alpha
19. Clarithromycin	19. Paliperidone palmitate	19. Epirubicin	19. Alteplase
20. Sertraline	20. Golimumab	20. Gemcitabine	20. Aflibercept
21. Citalopram	21. Sitagliptin	21.Rocuronium	21. Bendamustine
22. Alendronate	22. Vedolizumab	22.Risperidone	22.Vaccine: Rotavirus
23. Ranitidine	23. Fluticasone–salmeterol	23. Omeprazole	23. Abacavir–Dolutegravir: Lamivudine
24. Risperidone	24. Glecaprevir–pibrentasvi	24. Infliximab	24. Palivizumab
25. Interferon Beta 1A	25. Atorvastatin	25. Propofol	25. Factor VIII

Within the top 25 drugs purchased by inpatient settings in 2001, spending was greatest on erythropoietin alpha, at $91.2 million, followed by alteplase at $38.8 million, and cyclosporine at $32.6 million (Table 1). In 2020, the drug with the greatest spend in the inpatient sector was pembrolizumab at $361.6 million, followed by nivolumab at $253.0 million, and daratumumab at $218.0 million. Please see Additional file 1: Supplementary appendices for additional information.

### Drug categories of outpatient spending for top 25

**Fig. 2:**
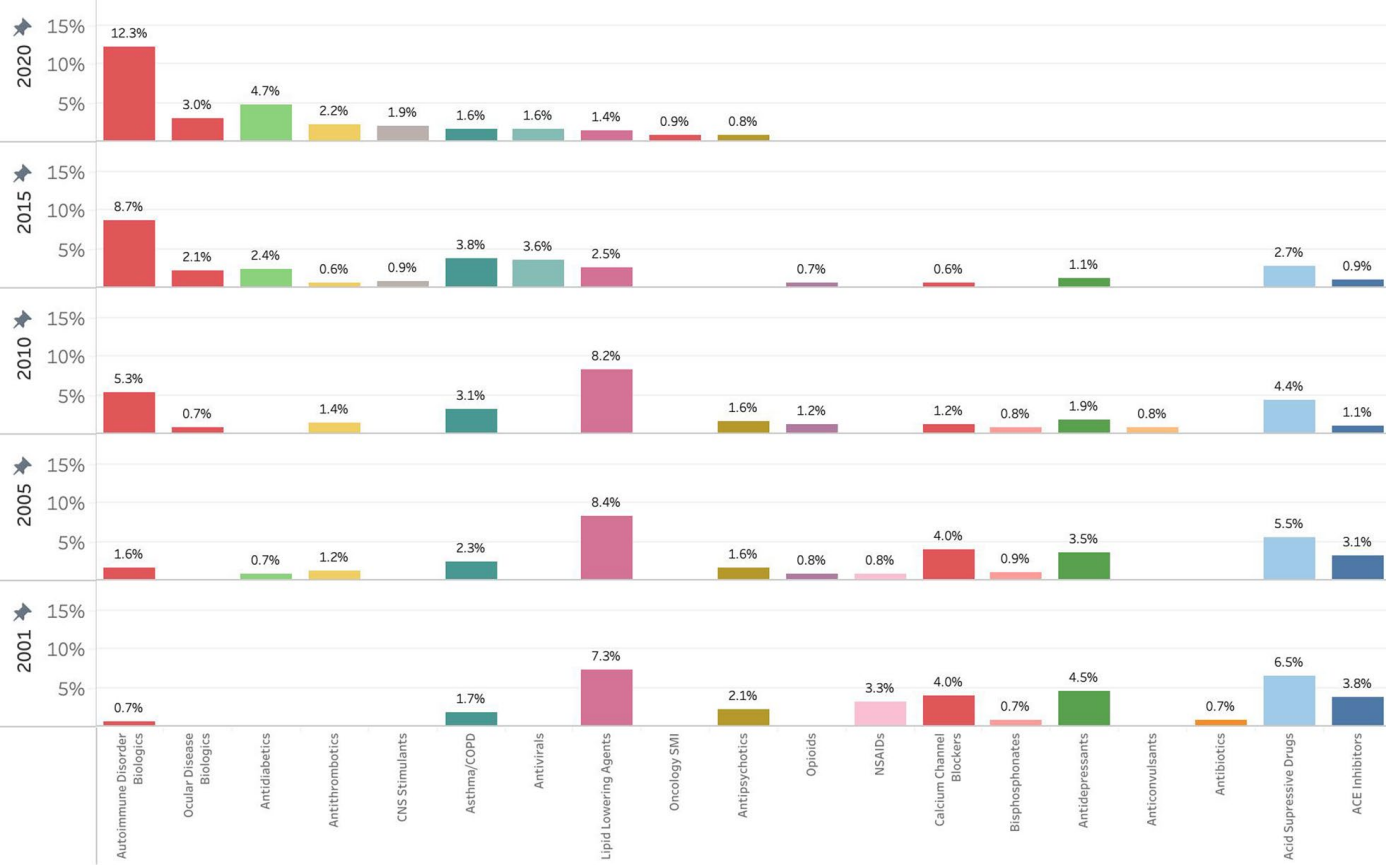
Top 25 drugs in drugstore purchases as proportion of total drugstore drug spending, categorized by class

In 2001, lipid lowering agents were the largest category of drug represented in the top 25 of outpatient purchasing and were closely followed by acid suppressive agents—which includes both proton pump inhibitors and H_2_ receptor antagonists (Fig. 2). In 2005, spending on biologics, which includes both autoimmune disorder biologics and ocular disease biologics, as a proportion of total outpatient spending was more than 10 times that of 2001. The proportion of spending for other categories, such as acid suppressive drugs, antidepressants, and calcium channel blockers remained relatively consistent. From 2005 through 2015, opioids make an appearance in the top 25 drugs defined by spend but are no longer present in 2020. From 2001 to 2020, there was a marked decrease in spending on lipid lowering agents, and an increase in antidiabetic agents. The proportion of total drugstore spending for biologic agents, increases from 2001 to 2020, by more than 2200%; with most of this growth attributed to autoimmune disorder biologics. In contrast to the rise in spending on biologics, the proportion of outpatient spending represented by the top 25 drugs has decreased by almost 5% from 2001 to 2020.

### Drug categories of inpatient spending for top 25

**Fig. 3:**
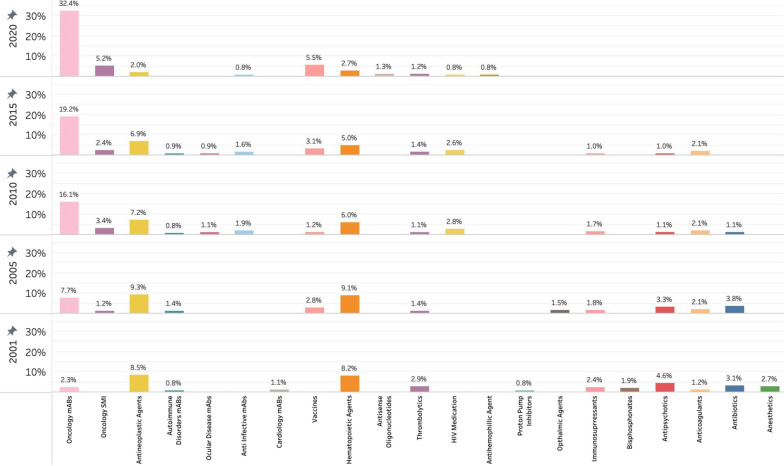
Top 25 drugs in inpatient purchases as proportion of total inpatient drug spending, categorized by class. mABs = monoclonal antibodies; SMI = small molecule inhibitors

In 2001, antineoplastics were the largest category of drug represented in the top 25 of inpatient purchasing and were closely followed by hematopoietic agents (Fig. 3). In 2005, the proportion of total drug spending on oncology monoclonal antibodies had more than doubled, with spending for other drug categories remaining relatively consistent. Spending on oncology monoclonal antibodies more than doubled again by 2010, with drug categories such as antibiotics, anesthetics, and thrombolytics seeing decreased spending as a proportion of total inpatient drug spending. From 2001 to 2020, there was a decrease in hematopoietic agents and antipsychotics, and an increase in spending on vaccines. Proportions of total inpatient drug spending in 2015 were similar to 2010, with a slight increase in the proportion of drug spending on oncology monoclonal antibodies. In 2020, the proportion of total inpatient drug spending due to oncology monoclonal antibodies was approximately 50% greater than in 2015. In 2001, the top 25 drugs purchased in the inpatient sector were captured by 13 drug categories. By 2020, the top 25 drugs represented an additional 12.4% of total inpatient drug spending and were captured by 10 drug categories.

## Discussion

Spending on the top 25 agents has become increasingly concentrated on fewer drug classes, which was more noticeable in the inpatient sector—nearly one-third of drug spending in the inpatient sector in 2020 was spent on biologics. In both settings, biologic agents make up the highest proportion of spending in the top 25 drugs. Although inpatient spending is dwarfed by outpatient spending, from 2001 to 2020, the average annual growth rate of 7.0% per year for inpatient drug spending is much higher than the 5.3% annual growth rate in the outpatient sector. Over the same period, the annual growth in the population of Canada has not exceeded 1.4% [7]. This highlights the need for a more effective, coordinated approach to cost-control mechanisms in Canadian inpatient settings.

Importantly, cost-control mechanisms are often specific to the sector in which the drug is sold. In outpatient settings, cost-control strategies for prescription drugs, like restricted benefits, co-insurance, deductibles, and annual or lifetime maximums, are well-developed but used infrequently [8]. Cost-control strategies for medications in inpatient settings are less widely understood in Canada. The American Society of Health-System Pharmacists (ASHP) has published the “ASHP Guidelines on Medication Cost Management Strategies for Hospitals and Health Systems” [4], but similar guidelines by the Canadian Society of Hospital Pharmacists do not exist. Although cost-control strategies are likely used in Canadian hospital settings, there is no unified compendium of cost-control strategies for prescription drug spending in the Canadian inpatient sector.

Annual growth rates for the top 25 drugs in each setting were of similar magnitude, with 8.5% for hospital purchasing and 4.4% for drugstore purchasing. The observed concentration of prescription drug spending on few agents suggests that successful cost-control mechanisms applied to few agents has the potential for large budget impact. Within the top 25 drugs ranked by spending, biologic agents stand out for their large market share. In both outpatient and inpatient sectors, the proportion of total drug spending for biologics has increased more rapidly than any other drug category. Specifically, this analysis highlights the need for cost management strategies applicable to biologic agents—in both outpatient and inpatient settings. These cost management strategies will likely differ from the payer-specific cost management strategies that have been used previously.

Although biologic drugs appear to have a natural monopoly at release, if present, this state does not necessarily last [9]. Since introduction of Neupogen® (filgrastim) in the United States, multiple competitors offering substantial discounts to the reference biologic have been introduced; which has resulted in a significant loss of market share for Neupogen [9]. This suggests that the adoption of biosimilar drugs could be used to reduce total spending for other biologic agents, in both inpatient and outpatient settings. Despite the availability of biosimilars for some of the most frequently used biologic agents, uptake and use of biosimilar agents in Canada has been slow [10, 11]. Compared to other Organisation for Economic Co-operation and Development nations Canada is a notably slow adopter of biosimilar agents [10]. Crosby et al. [11] estimates that a mandatory nonmedical switching policy for infliximab and etanercept alone across Canada would have resulted in nearly $240 million in cost savings in 2019. Adoption of biosimilars is not the only option to reduce spending on biologic agents. Other policy options include reduced entry costs of biosimilars, adjusting incentives embedded in payment policies, and revisiting allowable contracting strategies for biologics [9]. In coming years, Tadrous et al. [3] predicts downward pressure on spending due to generic formulations (e.g., recently available/forthcoming generic formulations for apixaban and rivaroxaban) and biosimilars (e.g., the biosimilar formulation for adalimumab approved by Health Canada in 2020 which was number 2 in outpatient spending). But given the increases in spending on biologic agents over the last 20 years, it is unlikely that current cost-control mechanisms will be sufficient.

This analysis has limitations. Spending data were considered in isolation without information about the payer, number of patients treated, or indications for treatment. Dataset used reflects only the prices paid by drugstores and hospitals. Some other factors that may influence drug use and expenditure include price changes, availability of generics, international prices, inflation, and entry of new drugs [6]. For drugs used in hospital, the price paid by the hospital also reflects the cost of the drug to a Canadian publicly funded healthcare system. For drugstores, the price paid by the drugstore imparts little information about the total cost of care and is independent of the ultimate payer. Additional markups and dispensing fees are often applied to prescriptions dispensed, and the cost to the payer is not well captured in the prices paid by drugstores. Furthermore, data were aggregated at the national level. Although it would have been interesting to compare spending patterns between provinces, drug price negotiations with manufacturers occur at the national level, and analysis of this data provides additional information about Canadian spending that could not have been captured with other data sources.

A unique strength of this IQVIA’s Canadian Drugstore and Hospital Purchases Audit data is the systematic inclusion of purchasing by both hospitals and drugstores, regardless of the ultimate payer for each drug. Differences in drug spending trajectories between inpatient and outpatient settings in Canada have previously been poorly understood. The Canadian Institute for Health Information publishes annual reports about prescription drug spending in Canada, but focuses on publicly funded prescription drugs, with nonuniform coverage of drugs used in hospital [1, 12]. This analysis shows that a greater proportion of total drug spending is going towards the top 25 drugs in the inpatient setting than in the outpatient setting; and in both settings, spending on biologic agents is growing. In data that do not include or stratify drug purchases by sector, these trends would have remained undetected.

## Conclusions

This paper describes spending trends for the drugs associated with the highest spending over time in both outpatient and inpatient sectors, with a shared increase in spending on biologics that spans both sectors. Identifying this concentration of spending on biologic agents is a key step in managing costs of prescription drugs in Canada. However, it remains to be seen if current cost-control mechanisms for biologic agents, such as increased use of biosimilars, will be effective enough to keep use sustainable. Future research efforts might focus on examining the effectiveness of cost-control mechanisms for biologic agents in Canada.

## Supplementary Information

**Additional file 1: Appendix S1. **Inpatient drug sales top 25. **Appendix S2. **Outpatient sales top 25.

## Data Availability

The datasets generated and/or analyzed during the current study are not publicly available due to their continued ownership by IQVIA Canada, but are available from the corresponding author on reasonable request.
