# Supplementary material for: Trends in Canadian prescription drug purchasing: 2001–2020
Source: J Pharm Policy Pract. 2022 Mar 17;15:20. doi: 10.1186/s40545-022-00420-4 (PMC8928614; doi:10.1186/s40545-022-00420-4)
Supplement: Supplementary file 1 — Additional file 1: Appendix S1. Inpatient drug sales top 25. Appendix S2. Outpatient sales top 25. [file 40545_2022_420_MOESM1_ESM.docx]

Appendix S1. Inpatient drug sales top 25

| **2001** | | **2005** | | **2010** | | **2015** | | **2020** | |
| --- | --- | --- | --- | --- | --- | --- | --- | --- | --- |
| **Drug** | **Sales** | **Drug** | **Sales** | **Drug** | **Sales** | **Drug** | **Sales** | **Drug** | **Sales** |
| Erythropoietin alpha | $91,178,231 | Rituximab | $92,630,590 | Rituximab | $161,668,865 | Rituximab | $196,460,494 | Pembrolizumab | $361,570,389 |
| Alteplase | $38,755,176 | Erythropoietin alpha | $88,981,314 | Trastuzumab | $146,934,509 | Trastuzumab | $185,085,663 | Nivolumab | $252,958,078 |
| Cyclosporine | $32,621,358 | Vaccine, pneumococcal conjugate | $51,823,468 | Bevacizumab | $85,345,806 | Bevacizumab | $101,060,419 | Daratumumab | $217,993,880 |
| Irinotecan | $30,448,929 | Irinotecan | $51,441,512 | Oxaliplatin | $85,006,136 | Oxaliplatin | $98,071,329 | Rituximab | $181,603,360 |
| Clozapine | $26,506,488 | Trastuzumab | $51,306,701 | Docetaxel | $70,720,679 | Bortezomib | $74,544,394 | Trastuzumab | $163,216,816 |
| Pamidronic acid | $25,229,243 | Darbepoetin alfa | $50,898,953 | Erythropoietin alpha | $65,966,505 | Pemetrexed | $68,492,177 | Pertuzumab-trastuzumab | $137,078,503 |
| Paclitaxel | $25,010,928 | Docetaxel | $40,754,999 | Darbepoetin alfa | $53,030,836 | Erythropoietin alpha | $60,924,476 | Ibrutinib | $112,681,112 |
| Olanzapine | $23,702,996 | Olanzapine | $33,157,473 | Bortezomib | $48,119,174 | Darbepoetin alfa | $57,030,782 | Durvalumab | $104,723,480 |
| Ciprofloxacin | $23,625,739 | Clozapine | $29,212,265 | Palivizumab | $46,938,338 | Vaccine hpv type 6,11,16,18 | $56,916,657 | Bevacizumab | $96,354,069 |
| Docetaxel | $19,766,636 | Filgrastim | $28,934,104 | Imatinib | $34,793,592 | Ipilimumab | $52,276,554 | Vaccine, pneumococcal conjugate | $87,296,479 |
| Filgrastim | $18,909,440 | Verteporfin | $28,494,325 | Enoxaparin | $30,208,019 | Pertuzumab-trastuzumab | $49,788,621 | Palbociclib | $74,652,436 |
| Ceftriaxone | $18,724,444 | Ceftriaxone | $26,208,991 | Vaccine hpv type 6,11,16,18 | $29,901,271 | Palivizumab | $48,319,063 | Haemagglutinin (non specific) | $72,728,489 |
| Enoxaparin | $15,949,737 | Tenecteplase | $25,831,540 | Filgrastim | $29,114,573 | Bendamustine | $44,774,832 | Darbepoetin alfa | $71,553,992 |
| Rituximab | $15,927,210 | Infliximab | $25,747,136 | Clozapine | $27,834,470 | Alteplase | $43,352,629 | Ipilimumab | $71,379,295 |
| Trastuzumab | $15,252,184 | Ciprofloxacin | $25,150,527 | Meropenem | $26,499,565 | Inactivated influenza virus | $38,093,456 | Osimertinib | $67,838,951 |
| Abciximab | $14,960,161 | Paclitaxel | $23,350,024 | Alteplase | $26,114,040 | Filgrastim | $35,292,136 | Nusinersen | $64,769,505 |
| Goserelin | $14,392,911 | Imatinib | $22,615,746 | Ranibizumab | $25,879,428 | Enoxaparin | $34,164,692 | Vaccine, hpv type-6,11,16, 18, 3 | $62,384,064 |
| Sevoflurane | $14,250,904 | Enoxaparin | $22,378,711 | Efavirenz-emtricitabine- tenofovir disoproxil | $24,075,440 | Clozapine | $30,782,519 | Erythropoietin alpha | $61,006,027 |
| Epirubicin | $12,948,677 | Gemcitabine | $21,877,643 | Emtricitabine: tenofovir disoproxil | $23,492,794 | Tacrolimus | $30,237,561 | Alteplase | $59,020,858 |
| Gemcitabine | $12,088,227 | Piperacillin-tazobactam | $19,246,529 | Tacrolimus | $21,449,472 | Dalteparin | $29,992,708 | Aflibercept | $49,213,706 |
| Rocuronium | $11,119,440 | Epirubicin | $18,453,181 | Dalteparin | $21,332,747 | Emtricitabine: tenofovir disoproxil | $28,248,920 | Bendamustine | $49,112,605 |
| Risperidone | $11,062,393 | Leuprolide | $18,196,830 | Leuprolide | $20,847,579 | Infliximab | $27,716,436 | Vaccine, rotavirus | $47,469,261 |
| Omeprazole | $11,055,743 | Cyclosporine | $17,348,087 | Infliximab | $20,481,267 | Ranibizumab | $27,001,842 | Abacavir-dolutegravir-lamivudine | $39,945,636 |
| Infliximab | $10,738,393 | Mycophenolate mofetil | $16,202,939 | Mycophenolate mofetil | $20,316,254 | Abacavir-lamivudine | $25,694,428 | Palivizumab | $39,639,915 |
| Propofol | $10,703,912 | Heparin | $15,982,255 | Atazanavir | $20,250,362 | Efavirenz-emtricitabine-tenofovir disoproxil | $23,728,956 | Factor viii | $39,324,727 |

Appendix S2. Outpatient sales top 25

| **2001** | | **2005** | | **2010** | | **2015** | | **2020** | |
| --- | --- | --- | --- | --- | --- | --- | --- | --- | --- |
| **Drug** | **Sales** | **Drug** | **Sales** | **Drug** | **Sales** | **Drug** | **Sales** | **Drug** | **Sales** |
| Atorvastatin | $467,042,211 | Atorvastatin | $926,893,420 | Atorvastatin | $898,058,516 | Infliximab | $897,971,365 | Infliximab | $1,233,935,545 |
| Omeprazole | $408,119,030 | Ramipril | $346,959,954 | Rosuvastatin | $645,124,467 | Ledipasvir-sofosbuvir | $617,664,132 | Adalimumab | $970,100,321 |
| Amlodipine | $207,948,299 | Amlodipine | $333,625,456 | Infliximab | $413,463,555 | Adalimumab | $579,432,242 | Ustekinumab | $527,810,031 |
| Paroxetine | $203,887,356 | Omeprazole | $300,100,977 | Fluticasone-salmeterol | $304,019,810 | Ranibizumab | $478,950,549 | Aflibercept | $522,913,776 |
| Simvastatin | $202,291,391 | Venlafaxine | $283,665,377 | Esomeprazole | $294,200,269 | Etanercept | $335,163,614 | Apixaban | $349,701,882 |
| Fluticasone | $183,165,966 | Pantoprazole | $252,879,371 | Clopidogrel | $281,366,172 | Fluticasone-salmeterol | $316,247,013 | Metformin-sitagliptin | $320,429,792 |
| Celecoxib | $182,312,445 | Olanzapine | $245,930,705 | Pantoprazole | $278,675,272 | Rosuvastatin | $288,091,467 | Semaglutide | $301,960,693 |
| Ramipril | $163,292,816 | Simvastatin | $190,783,137 | Etanercept | $271,942,788 | Atorvastatin | $275,156,686 | Ranibizumab | $301,492,979 |
| Rofecoxib | $160,796,330 | Clopidogrel | $184,013,232 | Adalimumab | $245,415,454 | Pantoprazole | $273,509,356 | Methylphenidate | $297,520,869 |
| Enalapril | $149,562,028 | Fluticasone | $177,710,213 | Amlodipine | $243,444,930 | Duloxetine | $256,686,198 | Etanercept | $286,159,405 |
| Olanzapine | $149,158,666 | Fluticasone-salmeterol | $170,433,444 | Oxycodone | $240,307,329 | Insulin glargine | $225,555,608 | Rivaroxaban | $272,302,154 |
| Venlafaxine | $116,830,476 | Rosuvastatin | $151,385,797 | Venlafaxine | $231,991,204 | Perindopril | $204,700,513 | Sofosbuvir-velpatasvir | $271,840,011 |
| Pantoprazole | $114,576,698 | Diltiazem | $145,590,543 | Ramipril | $218,502,433 | Esomeprazole | $200,001,317 | Budesonide-formoterol | $259,625,312 |
| Diltiazem | $109,209,352 | Esomeprazole | $141,719,596 | Omeprazole | $177,483,950 | Budesonide-formoterol | $191,635,023 | Empagliflozin | $256,387,093 |
| Pravastatin | $103,136,398 | Alendronate | $137,978,349 | Olanzapine | $177,280,740 | Methylphenidate | $191,339,188 | Lisdexamfetamine | $241,195,933 |
| Nifedipine | $102,605,016 | Lansoprazole | $135,246,478 | Pregabalin | $174,530,226 | Sofosbuvir | $181,869,634 | Insulin glargine | $238,962,564 |
| Lansoprazole | $93,781,520 | Nifedipine | $127,803,220 | Fluticasone | $167,724,123 | Tiotropium bromide | $179,597,533 | Ibrutinib | $237,298,370 |
| Lisinopril | $86,245,172 | Citalopram | $126,705,493 | Tiotropium bromide | $160,006,804 | Metformin-sitagliptin | $166,071,885 | Rosuvastatin | $228,483,742 |
| Clarithromycin | $78,139,325 | Paroxetine | $123,312,768 | Risedronate | $154,535,470 | Fluticasone | $165,804,393 | Paliperidone palmitate | $219,016,155 |
| Sertraline | $77,346,650 | Etanercept | $122,887,049 | Ranibizumab | $151,909,749 | Ustekinumab | $150,618,670 | Golimumab | $210,390,784 |
| Citalopram | $75,988,715 | Interferon beta 1a | $120,799,002 | Lansoprazole | $150,580,304 | Hydromorphone | $150,595,508 | Sitagliptin | $201,800,638 |
| Alendronate | $74,412,057 | Enalapril | $120,731,978 | Interferon beta 1a | $150,466,114 | Sitagliptin | $146,485,685 | Vedolizumab | $190,491,057 |
| Ranitidine | $71,919,408 | Oxycodone | $120,059,107 | Ezetimibe | $149,528,752 | Rivaroxaban | $141,933,402 | Fluticasone-salmeterol | $188,988,335 |
| Risperidone | $69,449,848 | Celecoxib | $114,046,704 | Citalopram | $148,928,475 | Aripiprazole | $141,305,001 | Glecaprevir-pibrentasvir | $175,055,487 |
| Interferon beta 1a | $68,624,654 | Rosiglitazone | $112,321,608 | Quetiapine | $148,555,602 | Amlodipine | $136,539,481 | Atorvastatin | $172,285,626 |
